# China’s policies: post-COVID-19 challenges for the older population

**DOI:** 10.1080/16549716.2024.2345968

**Published:** 2024-05-08

**Authors:** Xuezhi Wei, Guoqing Han, Quansheng Wang

**Affiliations:** Law School, Shandong University, Weihai, China

**Keywords:** Dynamic clearance, full liberalization, new coronavirus infection, vaccination policy, the elderly

## Abstract

On 7 December 2022, the State Council of China released ‘Measures to Further Optimize the Implementation of the Prevention and Control of the New Coronavirus Epidemic’. The previous three-year dynamic zero epidemic prevention policy was then replaced with a full liberalization policy. On 5 May 2023, the World Health Organization declared that COVID-19 no longer constituted a ‘public health emergency of international concern.’ However, given the ongoing prevalence of coronavirus, emerging mutations, and the liberalization of restrictions, there are increased risks of vulnerable people contracting new variants. Low vaccination coverage among older people with compromised immune systems, puts them at further risk. The policy shift will increase pressure on already stretched health infrastructure and medical resources. This short article adds to the current debate arguing that the Chinese government should take commensurate preventive measures, including strengthening medical facilities and equipment and targeting ongoing vaccination in older people.

## Abstract (plain language summary)

**Paper Context**
- The COVID-19 pandemic has disproportionately affected older people.
- This short paper discusses the consequences of China’s liberalization policy on the older population.
- It suggests that the Chinese government should have taken targeted measures to mitigate health risks occurring under the changed circumstances.

## Background

By the end of 2022, China’s three-year-old epidemic prevention policy of ‘dynamic zero’ had undergone significant adjustments. On 7 December 2022, the Comprehensive Group of the State Council Joint Prevention and Control Mechanism issued a notice on optimizing the implementation of preventive and control measures for the COVID-19 pandemic. The text describes a change from the previous ‘Notice on Further Optimizing the Preventive and Control Measures for the New Corona Pneumonia Epidemic Scientific and Precise Preventive and Control Work’ released on 11 November 2022. This change pertains to the policy for preventing and controlling the epidemic, which previously emphasized ‘dynamic zero’ but now supports ‘comprehensive liberalisation’.

Dynamic zero policy is a comprehensive control measure which aims to prevent the importation of cases from abroad as well as sporadic outbreaks within the country. The terms strict prevention and control and dynamic zero mean detecting and strictly controlling each case to prevent large-scale outbreaks. This approach does not aim for complete eradication of the virus but rather focuses on early detection and containment. Whenever a new COVID-19 case was detected, a total blockade of the affected area, including buildings and neighbourhoods, was enforced. These measures embodied a national commitment to prioritize people’s lives and well-being. These policies have prevented widespread infections and unnecessary mortality resulting from COVID-19 outbreaks.

Full or comprehensive liberalization assumes that there is no longer a need for strict preventive and control measures, such as regional blockades and personal isolation. Published research on the growth of new COVID-19 cases showed that interventions involving partial blockades are equally effective as full blockades in reducing the rate of disease growth [1]. In China, there has been no transitional phase between the policy of complete isolation and total blockade and the current policy of complete liberalization.

On 5 May 2023, the World Health Organization declared that COVID-19 no longer constitutes a ‘public health emergency of international concern.’ There may be some inevitable adjustments to the policy due to new developments and the direction of international epidemic prevention policy. China’s policy shift presents a huge challenge to ensure the health and safety of 267 million older people as the country moves to ‘full liberalization’.

One month after implementing full liberalization, China experienced waves of infection peaks. On 20 December 2022, the National Health and Wellness Commission reported that the cumulative number of people infected nationwide was at 248 million, translating to a cumulative population infection rate of 18% [2].

**Figure 1.**
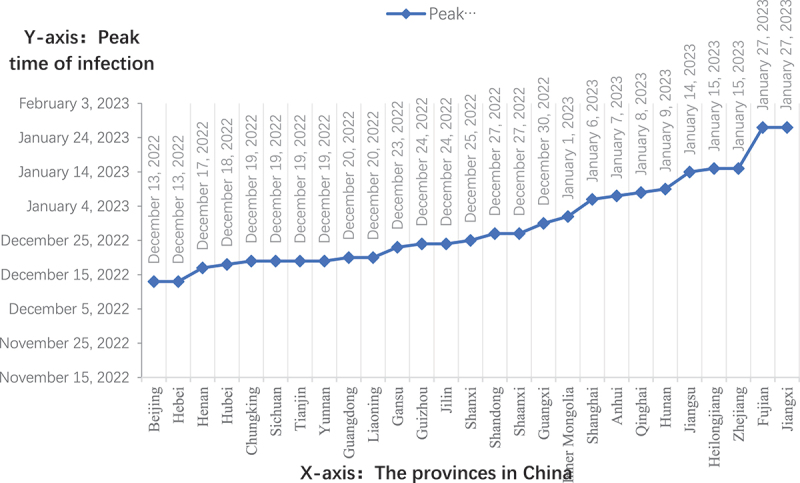
Peak time prediction for the first wave of infection post full liberalization in China.

**Note**: Big data experts predict the arrival time of the first-round infection peak across China through the Baidu search index. This prediction model has been verified in Taiwan, Hong Kong, and other regions and has certain referential significance. After the validation of the infection peak, the prediction model of the infection peak time is more accurate.

On 30 December 2022, Zeng Guang, chief scientist of epidemiology at the Chinese Centre for Disease Control and Prevention, reported that there were about 18 million newly infected people in Beijing. This was more than eighty percent of the population of Beijing at that time [3]. Scientists have extrapolated the time of the first round of peak infection in each province through the Baidu search index; see Figure 1 [4].

In December 2022, Wu Zunyou, the chief epidemiologist at the Chinese Center for Disease Control and Prevention, suggested that the 2022–2023 northern winter epidemic could be summarized as ‘one peak and three waves.’ The first wave was predicted to occur from mid-December to mid-January, mainly affecting urban areas. The second wave was expected to occur late January to mid-February 2023, associated with the movement of people for the Spring Festival. The third wave was expected to occur from late February to mid-March, the time when people generally return to work after the Festival. It is important to note that these predictions were based on a fast evolving situation. During the winter season, the new coronavirus epidemic was expected to peak in three waves [5].

**Figure 2.**
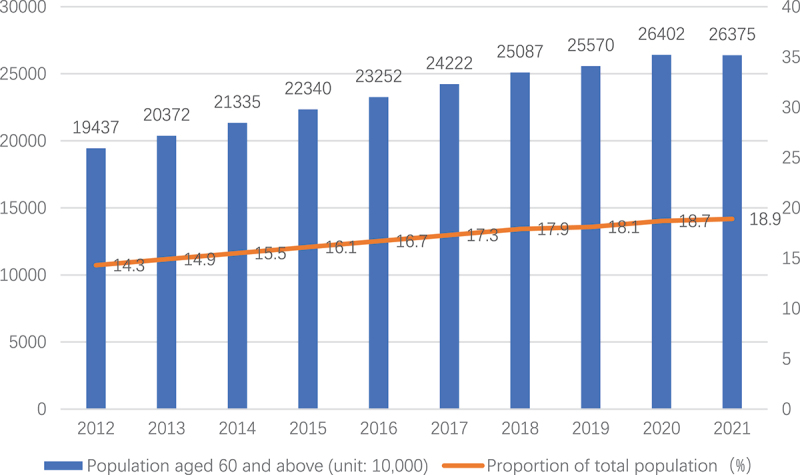
Number and proportion of the population aged 60 and above in China, 2012 to 2021.

The proportion of older people in China is growing rapidly. Population aging is becoming increasingly apparent. By the end of 2021, the number of people aged 60 years and older was 267 million, comprising almost nineteen percent of the entire population. See Figure 2.

With mass population infections, the older population is hardly an exception. Compared with younger people, disproportionately more older adults have vulnerable health and weakened immune systems. Additionally, relatively more older people have underlying health conditions such as diabetes, pulmonary embolism, or hypertension that can be complicating co-morbidities. Older people are therefore more at risk for coronavirus and other infections. China News Weekly published an article on 29 December 2022, titled ‘Be Alert to Viral Pneumonia Caused by Omicron; Critical Beds Near Capacity in Many Places.’ The article highlights the significant pressure on hospital facilities due to the high proportion of older people with serious illnesses, many of whom required admission to intensive care units [6].

Enumerating numbers of infections in the entire population and the number of conditions in the older population is challenging due to the lack of precise detection methods and statistical techniques. According to China’s Seventh National Census in 2021, there were 267 million individuals aged over 60 years. It was estimated that the number of COVID-19 infections in this age group would exceed 130 million. Fifty percent of the total population [7].

There is an ongoing shortage of necessary emergency medications and equipment. Pharmacies have not always been able to provide essential medicines. Moreover, some health facilities cannot meet basic treatment needs, hospitals are overcrowded, and more doctors and nurses are needed. Inpatient and intensive care units struggle to meet demands. The situation is particularly dire for older people and those in rural areas.

Although some progress has been made, voluntary vaccination against COVID-19 among older people has been progressing slowly. According to the National Disease Control Administration, in 2021 over 215 million people aged 60 and above had received at least one dose of the vaccine and of those, 206 million had completed their vaccination course [8].

On 20 December 2022, the National Centre for Health Statistics established objective criteria for defining COVID-19-related deaths. They include death, where pneumonia and respiratory failure are the primary causes of death. Deaths caused by other underlying diseases, such as cardiovascular disease or heart attack, are not classified as COVID-19 deaths [9].

Between 8 December 2022, and 12 January 2023, medical institutions across China reported 59,938 deaths related to COVID-19 infection. Among them, 5,503 were fatalities due to respiratory failure caused by COVID-19. An additional 54,435 deaths were attributed to underlying diseases in combination with COVID-19. The average age of those who passed away was 80 years, and ninety percent were 65 years or older. Approximately, fifty-six percent were aged 80 years or older. Underlying diseases, primarily cardiovascular disease, advanced tumours, cerebrovascular disease, respiratory disease, metabolic disease, and renal insufficiency, accounted for about ninety percent of deaths. Some suggest that the official reported death rate of COVID-19 may be significantly lower than the real death rate [10].

Countries worldwide are adjusting their policies to prevent and control the outbreak of the new coronavirus. While full liberalization is the current policy in China, relaxation of epidemic prevention and control measures cannot completely eradicate the virus. The risk of contracting the virus persists, and even those who have previously been infected cannot be certain of avoiding subsequent infections. Repeated infections can negatively impact on an individual’s physiological function, and can be particularly serious in vulnerable older people. The government has a responsibility to provide targeted measures to prevent further infections. The following are some possible strategies.

The first is to implement a comprehensive prevention policy to avoid primary or secondary infection particularly in the older population. Measures could be taken in key locations, such as nursing homes. Traditional Chinese medicine can be utilized for prevention, and herbal medicines can be given to older individuals to prevent infections and promote preventive care.

The government is responsible for ensuring an adequate supply of medications. A further suggestion is to encourage pharmaceutical companies to produce sufficient supplies mitigate hoarding, maintain order in the drug market, and provide free essential medicines to those in need.

A third strategy is to establish a classification and treatment system. The National Health and Wellness Commission and the State Administration of Traditional Chinese Medicine issued the COVID-19 Treatment Plan (Trial Version 9) in 2022 [11]. The plan requires proper case classification and treatment to ensure that patients with pneumonia symptoms and underlying conditions, as well as older individuals, are admitted to designated hospitals for centralized treatment. This plan should be expanded to include specialized care for critically ill older people with underlying diseases.

The fourth suggested strategy is to increase vaccination efforts in older individuals. Although the first-dose vaccination rate for individuals over 60 years has exceeded ninety percent, complete vaccination and booster immunization in older people is critical. The time interval between the first dose of booster immunization and complete vaccination in China exceeds three months in the older population. This interval should be reassessed for older people [12].

The fifth strategy is in regard to improving the organization of grassroots prevention and control efforts. The committee for urban and street residents, the committee for rural villagers, and a central grassroots prevention and control work team implemented the sub-piece (grid) using information technology and other means. The idea is to collect and manage fundamental health information for senior citizens residing in both urban and rural areas. This strategy provides a basis for better coordination of medical personnel, medications, referrals, patient transportation and epidemic prevention and control.

This ‘Current Debate’ article does not provide a comparative cross-country analysis. Future research on this topic is needed to better understand the impacts of epidemic prevention and control policies in different settings.

## Conclusion

COVID-19 has been a public health emergency at the international level. China will no doubt continue to modify the country’s epidemic prevention and control policies. The major changes in late 2022 have impacted the health of older citizens. Major public health policies require an evidence-based risk assessment prior to their implementation.
